# Effect size of contributory factors on adverse events: an analysis of RCA series in a teaching hospital

**DOI:** 10.1186/s40200-016-0249-3

**Published:** 2016-07-28

**Authors:** Zhila Najafpour, Mohamadreza Jafary, Morteza Saeedi, Alireza Jeddian, Hossein Adibi

**Affiliations:** 1 Health care management, Department of Health Economics and Management, School of Public Health, Students’ Scientific Research Center, Tehran University of Medical Sciences, Tehran, Iran; 2 Shariati Hospital,Tehran University of Medical Sciences, Tehran, Iran; 3 Emergency Medicine research center, Shariati hospital, Tehran University of Medical Sciences, Tehran, Iran; 4 Digestive Diseases Research Institute (DDRI), Tehran University of Medical Sciences, Tehran, Iran; 5 Health services management, Endocrinology & Metabolism Research Center, Endocrinology and Metabolism Clinical Sciences Institute, Tehran University of Medical Sciences, Tehran, Iran

**Keywords:** RCA, Contributory factors, Adverse events

## Abstract

**Background:**

One of the most important concerns of health care systems in the world is the patient safety issues. Root Cause Analysis is a systematic process for identifying root causes and contributory factors of problems or events. The objective of this study is to review RCA reports to determine the effect size of contributory factors on adverse events through an organizational perspective.

**Methods:**

This study was conducted in a tertiary care teaching hospital in 2014. The process of root cause analysis was taken from National Patient Safety Agency framework. We calculated descriptive statistics to determine the frequency distribution of contributory factors on each adverse event.

**Results:**

Having the process of 16 adverse events reviewed, 38 care or service delivery problems were identified which showed that 317 contributory factors and underlying causes had led to these problems. Accordingly, the most important contributory factors included the following: Task factors (20 %), education and training factors (16 %), communication factors (14 %), and team and social factors (13 %).

**Conclusions:**

RCA is an effective method of problem solving used for identifying the root causes of initial errors and finding ways to prevent the recurrences. In this study, lack of effective communication skills of nurses and other clinical staff when interacting with colleague and communicating with patients, failure to comply with health care provision standards, lack of adequate supervision on implementation of clinical guidelines and issues related to the organizational culture were the main determining factors which have been considered for implementing preventive measures with regard to the hospital specifications.

## Background

Patient safety is a worldwide issue which affects quality of care, clinical outcome effectiveness, patients’ quality of life, patient satisfaction, and savings of financial resources [1–3].

The first principle in medicine is “not to harm the patient”, thus avoiding and eliminating injuries and harms to patients is becoming a main concern in health care systems [4]. Approximately, one out of ten patients admitted to hospitals experiences injury or medical error during their staying in hospital which may result in mortality and morbidity of patients [5].

The Institute of Medicine (IOM) reported that medical errors lead to nearly 100,000 patient deaths per year. Moreover, an Australian study estimated that cost of adverse events is accounted $4.7 billion a year [6] and it caused patient safety to become an increasingly high priority in health care systems in 1999 [4].

Typically, medical errors are result of a combination of contributing factors such as poor communication, health care system weaknesses, staff’s lack of education, etc; rarely there is just one causal factor [7].

A new approach to medical errors is shifting responsibility from individuals toward the system. Hospitals should investigate sentinel and serious events and analyze their root causes [8]. There are several methods, including reactive and proactive methods, for clinical risk management which enhance the quality of care and guarantee patients’ safety [9]. Root cause Analysis (RCA) is a reactive method for investigating an event and finding its underlying factors. In addition, RCA is an answer to the questions about what, how, and why an event happened, and is also used to design preventive interventions [7]. From the mid-1990s, RCA has been introduced and used for improving patient care and safety. It is used for learning from mistakes and preventing future errors or injuries by identifying and removing the root causes of errors [10].

The National Patient Safety Agency (NPSA) framework has outlined a process of investigation and analysis of clinical incidents and has provided recommendations for action as well. In this framework, factors influencing clinical practice include nine categories of patient factor, task factor, individual factor, communication factor, team factors, education factor, equipment and resource factor, work condition factors and organizational & management factors [11].

Therefore, the objective of this article was to review serial RCAs and reveal the distribution of contributory factors on adverse events.

## Methods

A retrospective qualitative study using a RCA methodology was undertaken in a 600 bed tertiary care teaching hospital in 2014 in Tehran, Iran. All cases were assessed by a fixed team in a 15-month period. The process of RCA was taken from the National Patient Safety Agency (NPSA) for the current study [12]. Contributory factor classification framework included the following 6 steps:

1. Identification of events was done by a voluntary adverse events reporting system including electronic and paper form for all events (near misses, adverse and sentinel events). Incident forms were completed by frontline staff and delivered to safety office of clinical governance committee. Moreover, suspicious cases that were reviewed in mortality committee were referred to RCA team for further consideration. Following the previous literature, we defined an error as a mistake: either a failure of a planned action to be completed as intended (i.e. an error of execution), or the use of a wrong plan to achieve an aim (i.e. an error of planning)” [2].
  In this study, we chose cases of death or serious injury to patients and in situations where such events were narrowly avoided based on two physician’s opinions [13].
  We asked for a confidence score from the reviewing physician to indicate the presence of an adverse event or near miss, and then these cases entered the study in form of RCA in order to be assessed by the team. Preventability was then independently judged by two investigators. Accordingly, an adverse event was considered preventable if it was avoidable by any means currently available unless that means was not considered a standard care. We focused on learning potential and preventable harm, but estimation of preventability is not an exact science [6]. we classified the severity of harms according to the National Council for Medication Error Reporting and Prevention Index [14].
2. Team member selection: all of the RCA were performed by a team whose fixed members consisted of people with various backgrounds and different levels of experience (chief executive, patient safety coordinator, patient safety consultant, supervisor and head nurse, matron of the hospital). Other members were interviewed in accordance with the subject under investigation. The participants of the team all attended a one-day course on RCA.
3. Data collection was performed through conducting interviews with involved staff, examination of medical records along with probing policies, guidelines and other related documents. Face-to-face semi-structured interviews were conducted with patients and their family. The primary purpose of this interview was to establish a chronology of how error developed from patient’s opinion.
4. Determining the incident chronology: team mapped incidence based on timeline of events and event flow diagram was constructed. An event flow diagram is a chronological diagram of the series of events leading up to an adverse event. The team also identified who needed to be interviewed in order to obtain this information. RCA requires rich and detailed data in order for ‘root causes’ to be identified accurately. Therefore, additional interviews were also conducted with physicians, nurses and other related people. These interviews were conducted either face-to-face or over the telephone depending on the preference of the interviewee.
5. Identifying problems related to delivery of medical cares were performed by RCA team members. These problems consist of all errors such as failure to monitor and observe along with failed medical procedures that take place during medical care delivery. They were identified during error analysis and service provision processes, decisions, and procedures. Furthermore, the question ‘why’ was asked repeatedly until the root cause of the admission was identified.
6. Developing action plans: in this step, in order to avoid and eliminate the system weaknesses that have been revealed, the group formulated a set of recommendations and action plans.

In this survey, RCA team investigated 16 adverse events that were referred and were likely to recur based on team’s opinion. Having all required data gathered, the group had a meeting about the whole process. Then the group defined a timeline for the event, and used brain storming method and fishbone diagram by which care and service delivery problems, contributory factors and preventive measures were determined.

Regarding ethical and legal considerations, the investigated reports in this article stayed unnamed and with no mention of specifications.

Descriptive statistics were used to summarize the data and to determine the frequency of distribution of contributory factors on each adverse event by SPSS software, version 12, and the synthesized data were translated with a fishbone diagram to identify root causes of the problem.

We examined the characteristics of errors and the frequency of contributory factors as a cause of the errors and then the contribution of each factor to all factors affecting the occurrence of event was determined.

## Results

In this study, 16 cases were reviewed and their reports were published.

Table 1 shows a descriptive summary of each event. As shown below, there are some similar events such as 2 cases of bed fall, 3 cases of unsafe patient transfer, 2 cases of transfusion error and 4 cases of delayed treatment (Table 1).

**Table 1:** Excerpt from root cause analyses reports

NO	Event	Outcome for patient	Descriptive Summary
1	Preventable abortion	Fatal death	After admission of a 32-week pregnant woman in emergency with probability of premature delivery, the patient decided to leave the hospital because of lack of NICU bed the patient loses her fetus due to seizure beyond the hospital.
2	Transfusion error	Transferred to the intensive care unit. After 2 days when the liver enzymes were normal, he was discharged.	Two patients with similar names were admitted to the emergency ward. When the blood bag was received by the ward, the patient’s nurse miscalled the patient who had an order for transfusion (…) and relief nurse completed the mistake
3	Hypoglycemia that led to death	Death	During the transmission of a diabetic patient from emergency to general ward, the evaluation of clinical situation and blood glucose levels were missed for many hours, the patient arrested and finally passed away.
4	Transfusion error	Hemoglobinuria, tachycardia	The nurse took sample for blood cross match from wrong patient and therefor an inappropriate blood bag was sent to the ward (…) the patient asked the nurse for blood group mismatch but got no response
5	Unsafe patient transfer	Death	The patient was transferred to imaging ward with unstable clinical situation (few minutes after cardiac arrest and CPR) and (…) two hours later, the patient arrested again and unfortunately …
6	Bed fall	Suture in the elbow	After discharging from emergency ward, the patient falls when his family left him alone for asking physician to visit patient again
7	Bed fall	Pain and bruising	The complicated patient falls when he was left alone in sonography room on the bed without bedside
8	Unsafe patient transfer	Death of patient	The multiple trauma case was transferred to imaging ward with a helper and in CT room the patient arrested (…)
9	Unsafe patient transfer	Death of patient	The patient with cardiac and renal problems was transferred from emergency to general ward with a helper, meanwhile, seizure happened for patient and he entered the ward with cyanosis and cardiac arrest
10	Delayed treatment	Death of patient	After evaluation of patient and decision for therapeutic abortion in cat lab conference, the plan was postponed because of need for some consultations and (…) 4 days later, the patient died with cardiac arrest tableau
11	Delayed diagnosis and treatment	Death of patient	The diagnosis of patient’s problem was not described sufficiently and she was undecided between two medical teams, even so when patient was transferred to operation room, her attendant did not approve consent form (…)3 days later, the patient died with pulmonary and cardiac arrest.
12	Wrong surgery	Reoperation	The nurse misunderstood the doctor handwriting and during telephone conversation, the patient was transferred to operation room and underwent a wrong surgery
13	Resuscitation failure	Death of patient	The CPR trolley was not renovated after resuscitation of previous patient at last night. The suction unit and ambo bag were not stand by and monitoring unit was transferred to another ward
14	Delayed diagnosis and treatment	Death of patient	There were no clear decisions from the two medical teams for patient management. Moreover, The ordered decisions were only recorded and not been performed for 3 h (…) unfortunately the patient expired in ketoacidosis state
15	Cautery burning	Sore in buttock area	The patients who were getting CABG complained from skin problems and examination suggested cautery burning especially in buttock area
16	Delayed treatment	Death of patient	The involved parties did not follow treatment plan of therapeutic abortion and the patient left the hospital without any action (…) 2 weeks later, the patient returned with critical conditions and medical care were ineffective.

In studied cases, 57 % of investigated patients were female and the rest were male. Patients aged between 31 and 78 years old and 13 % were hospitalized in internal wards, 50 % in surgical wards and 37 % in emergency ward.

Out of investigated cases, eight led to death, six led to undesirable outcome, and two were near misses.

Multiple interviews with different individuals involving 5 cases with patients and their families, 9 cases with nursing personnel, 12 cases with physicians and their assistants, and 7 cases with other service providers including medical equipment unit, patient helpers, and other departments were used to gather information.

In reviewing process of 16 adverse events, RCA team defined 38 care or service delivery problems and thereafter the team revealed 317 contributory factors and underlying causes for these problems (Table 2).

**Table 2:** Frequency of contributory factors in the 16 RCA cases (PERCENT^a^)

Event	Problem	Patient	Employee	Task	Communication	Team	Education	Equipment	Organization	Environment
Delayed treatment	Not emphasis on serious following up, convincing and admitting of patient.	7.69	7.69	34.62	15.38	19.23	11.54	0	3.85	0
	The high risk patient left the hospital and therefore therapeutic abortion was postponed									
	Unplanned and precocious extubation (early extubation)									
	Not recording CPR documents									
Delayed diagnosis and treatment	Not checking orders of internal medicine team	5.88	17.65	11.76	11.76	5.88	29.41	0	5.88	11.76
	delay in following up the diabetic ketoacidosis									
Delayed diagnosis and treatment	The diagnosis and treatment plan for patient were delayed.	17.6	5.88	17.65	11.76	11.76	11.76	11.76	5.88	5.88
Delayed treatment	Many bugs in the consulting process (subject of consultation, unnecessary consultation, typical response to consultation)	4.35	4.35	30.43	8.70	21.74	17.39	0	8.70	4.35
	lack of effective communication between cardiology and gynecology teams for clinical decision making									
Hypoglycemia that led to death	Medical and nursing staff did not follow the patient’s blood glucose status	3.70	14.81	14.81	14.81	14.81	7.41	0	11.11	18.52
	Not assessing the patient’s clinical condition (before and after transfer)									
	Many bugs in patient transfer process from emergency to general ward									
Resuscitation failure	The resuscitation trolley was not checked and renewed after use	0	4	12	8	8	16	20	16	16
	The CPR team not being well-organized for controlling problems									
	Lack of intact(necessary) equipment									
Unsafe patient transfer	Unsafe patient transfer process (ward to ward)	0	0	23.08	7.69	7.69	30.77	15.38	7.69	7.69
	Unsafe patient transfer using regular ambulance									
Unsafe patient transfer	transferring patient without observation of a nurse or physician	0	0	16.67	16.67	0	33.33	0	0	33.33
Unsafe patient transfer	transferring unstable patient to imaging ward	8	16	16	16	12	24	0	4	4
	responding to medical consultation by junior assistant									
Bed fall	pull down of bedsides and transferring patient by his attendant	18.75	6.25	25.00	6.25	6.25	18.75	0	6.25	12.50
	Delay in patient visit and missing the necessary assessment before discharge									
Bed fall	lack of classification of falling risk assessment for high risk patients	0	0	15.38	15.38	7.69	23.08	7.69	15.38	15.38
	Poor patient care during the process and waiting time for para clinic measures (imaging)									
Transfusion error	Patient identification error	15	10	15	10	15	15	5	5	10
	not execution of blood transfusion protocol									
	miscommunication between nurse and patient									
Transfusion error	employing inappropriate personnel in emergency ward	11.54	11.54	15.38	7.69	15.38	11.54	3.85	3.85	19.23
	disregarding safety of blood transfusion process									
	incomplete information on the blood bag									
	blood transfusion by nursing student as a relief									
Wrong surgery	error in surgery plan for patients	0	5.56	27.78	33.33	11.11	16.67	0.00	5.56	0
Cautery burning	burning with cautery instrument in heart surgery patients	20	10	30	10	10	10	10	0	0
Preventable abortion	non-admission of high risk patient	11.43	11.43	11.43	20	17.14	8.57	2.86	11.43	5.71
	not accurate assessment of the patient									
	allowing patient to leave hospital without warning to supervisor and chief resident									
	lack of coordination in NICU admission of other sites									

^a^The frequency of each factor to total factors identified for each event

Distribution of these factors in seven groups of Contributory Factors Classification Framework, which is described by National Patient Safety Agency, is shown in Table 1. Accordingly, the most important contributory factors included task factors (19 %), education factors (16 %), communication factors and team factors (each one 13 %) (Table 3).

**Table 3:** Contributory Factors Classification Framework, frequency and proportion

NO	Category	Frequency	% of total (n)
1	Patient factors	25	7.89
2	Individual (staff) factors	28	8.83
3	Task factors	61	19.24
4	Communication factors	43	13.56
5	Team factors	41	12.93
6	Education Factors	51	16.09
7	Equipment and resources	14	4.42
8	Organizational factors	24	7.57
9	Working condition factors	30	9.46
10	Total	317	100

Due to the classification of similar events, task and team factors had more significant effect on 2 cases of transfusion error. In the same way, task and education factors highly affected 4 cases of delayed treatment, education and task factors affected 3 cases of unsafe transfer, and, finally, task and education factors had more impact on 2 cases of bed fall.

## Discussion

In this study, the information obtained from 16 RCA cases was analyzed by a single RCA team. 20 contributory factors for each event and eight factors for each service or care delivery problem were identified.

Task factors had the most prevalence with 20 % of total contributory factors of adverse events; however, the rate increased to 25 % in patients who suffered from diagnosis and treatment errors. Various factors including lack of a precise framework for specification and structure of responsibilities, no access and not utilizing instructions and decision making aids have led to defects in this field. Moreover, it has not been a long time since governing organizations have focused attention on the matter of clinical guidelines, and it is a tangible fact that there are no reliable guidelines and instructions based on the evidence and practical aspects for many trends and services. Another concern was the lack of proper definition of supervision procedure on guideline-based functions of providers, particularly in teaching hospitals, which has resulted in passive approach to clinical guidelines and instructions.

The contribution rate for education and training factors in the occurrence of events was 16 %, while the rate increased to 27 % for the occurrence of unsafe patient’s transfer faults and 20 % in the occurrence of falling events.

Adverse events (AEs) might occur in hospital transports; our results indicated that the most common error resulting in an event was related to equipment malfunctions and failure in transferring the patient. Sometimes the patient’s condition is deteriorated as a consequence of disease progression. In a study from the United Kingdom, 56 children were prospectively monitored for AEs during inter-hospital transport. Seventy-five percent of the patients experienced important complications, with 20 % of those being life-threatening. Careful planning, monitoring, and resource allocation such as assigning appropriate personnel for the transport, are extremely important to ensure that patients remain as safe as possible [15].

The contribution rate related to communication factors was 14 % which increased up to 33 % for wrong surgery event and 20 % for preventable abortion. Communication error exists between various groups of staff, but this is more probable among physicians and nurses who are mainly responsible for taking care of patients [16]. Organizational procedures and guidelines determining what information by who and when should be transferred are of great significance [17]. Different providers (physician, nurses, students, etc.) have to be trained for communication skills and safe information transfer, particularly at the time of handover, and also for how to transfer a telephone order. Imperfect education of inter-professional and staff-patient communication skills are the main threatening factors for patient safety issues [18]. Numerous studies have made the effects of this failure equal to failure in accessing patient safety [19]. A study by Gawande considered that lack of effective communication among the personnel was responsible in 43 % of analyzed errors [20].

This failure is more profound in communication between physician and nurse in inter-professional communications. In Smith’s study, 91 % of reported medical faults were due to this communication failure. He has also pointed out that failure in inter-professional and staff-patient communication were to blame in 66 % of adverse events from 1995 to 2004 especially in wrong site surgery and medical errors [21].

In Greenberg’s study, the most failure was related to poor pre-operation communications with patients and lack of proper verbal communications which frequently occurred at the time of patient’s handoff or transferring him/her to the new place (ward or OR) [22].

In our study, the failures in transferring information, especially at the patient delivery, were the main contributory factors. Other responsible factors included communication skills defect, language barrier and lack of nursing staff. Also, unfamiliarity of the staff in various wards with transferring information had a role in the occurrence of adverse events.

Transitions from one care provider to other put patients at increased risk of injuries and errors. A standardized approach to hand-off communication helps minimize these risks. Patient report from nurse to nurse needs to be a sophisticated, precise, comprehensive compendium of patient information that focuses on patient safety. One recognized approach to addressing this concern is the SBAR (i.e. situation, background, assessment, recommendation) communication technique. Reference cards with the SBAR communication approach can be used by all staff members during hand offs in the preoperative, intraoperative, and postoperative phases of surgical patient care [23].

The contribution of team and social factors in analyzed events was approximately 13 % which increased to 17 % in the case of preventable abortion and 16 % in the case of diagnosis and treatment errors. Failures in communication within inter-professional healthcare teams are established causes of medical error and negative health outcomes. Emphasizing on inter-professional teamwork principles and effective team communication has a great significance in improving patient safety [24, 25].

In addition to reducing faults and stress, sharing experiences and learning are among the advantages of teamwork in clinical practice which can have a tremendous influence on improving patient safety [26]. However, there are numerous challenges in human and personality relations, so the first suggested solution is improving the workplace culture. The results of Mazzoco’s study indicated that the patients whose operation team had a weaker teamwork were more exposed to death or side effects. In other words, weak teamwork influences the occurrence of adverse events [27]. This has also been confirmed in Giardiano’s study which indicated that the teamwork problems related to coordinating and decision making were the main contributory factors in diagnosis and treatment delay and errors [28]. One of the requirements to promote teamwork and team-based treatment is promoting inter-professional communication skill that should be considered in continuous training system and medical science education [29].

Consistent function of the health care teams can result in better clinical outcomes and patient’s satisfaction, and, finally, improves patient safety consequences. Training can be effective as a strategy for improving the function of the Clinical team by concentrating on leadership, conflict resolving, and adapting to changes and teamwork [30].

Our study had several limitations that its main limitation was gathering the reliable data for root cause analysis. So, we were supposed to collect the necessary data from any possible ways including patient records, interview with involved personnel, attending viewpoints, inspection of event location and patient and his attendants meetings.

## Conclusion

In this study, the service and care delivery problems were attributed to individuals’ preferences for individual activities and individualism instead of team work as an organizational culture. In addition, inability of coordinators to control and guide service and care providers due to weak leadership skills is of great significance. Lack of complete briefing of team members about their positions and duties in the health care team are examples of other problems. Inappropriate attention to clinical guidelines and instructions in the academic training process of medical students and nurses, lack of enough continuous education programs and lack of serious concern about patient safety by hospital authority as well as unsuitable implementation of safety protocols were the most important causes affecting the occurrence of errors extracted from focus group discussions. Because of the discrepancy in designing and executing preventive measures, it seems that the evaluation of distribution of contributory factors is important. Collecting further RCA samples and information in future studies can improve this evaluation. However, the differences and level of hospitals and providing centers and the effects of such differences on the way of offering services should be considered for real conclusion and policy making.

## Abbreviations

CDP, care delivery problems; NPSA, National Patient Safety Agency; RCA, root cause analysis; SDP, service delivery problems
